# Editorial: Bridging Cognitive Neuroscience and Neurosurgery for Effective Brain Mapping

**DOI:** 10.3389/fnhum.2022.899341

**Published:** 2022-04-12

**Authors:** Elena Salillas, Alessandro Della Puppa, Carlo Semenza

**Affiliations:** ^1^Department of Psychology and Sociology, University of Zaragoza, Zaragoza, Spain; ^2^Neurosurgery, Department of NEUROFARBA, University Hospital of Careggi, University of Florence, Florence, Italy; ^3^Department of Neuroscience (Padova Neuroscience Center), University of Padova, Padua, Italy

**Keywords:** cognitive neuroscience, neurosurgery, brain mapping, language, memory, attention

The present topic groups studies with the common goal of filling the gap between cognitive neuroscience and brain surgical mapping. The selected contributions imply originality in their approach to surgery. Far from exhaustive, they exemplify what can be done through cognitive-inspired methodologies. The studies combine very diverse techniques and specific aims. They stress the need of adjusting the commonly used tasks (i.e., addressed cognitive processes) during peri-surgical mapping. They involve neuroimaging paradigms that include an assessment of the inter-subject variability, allowing a transfer from group to the single case level. They also stress the need for convergent validity across techniques and the longitudinal evaluation of functions. A very insightful revision paper is included on brain mapping in multilinguals.

Three of the papers use navigated transcranial magnetic stimulation (nTMS). Preoperative nTMS can be a useful technique, especially in those cases where awake surgery is not feasible (i.e., age, comorbidities; Rath et al., 2014). Senova et al. provide a pipeline for gaining precision in the application of presurgical mapping to surgery. Their case study reports a right frontal tumor patient assessed pre-surgically using resting-state fMRI (rs-fMRI), tractography, and motor nTMS mapping. Despite language functions were not addressed using nTMS, the motor nTMS mapping and neuroimaging data allowed for correction of brain shifts intraoperatively. This was achieved thanks to intraoperative CT (iCT) at key surgical moments with an expected brain shift.

We included two other nTMS papers which do not involve patients, but with methodological and task proposals that might prove useful for presurgical language mapping. Ohlerth et al. propose a refinement of two frequently used tasks during peri-surgical mapping: action naming and object naming (Ruis, 2018). In particular, the paper determines the brain sites causally linked to those functions using nTMS and uses positive sites as ROIs for tractography. Both tasks were equally sensitive for visualizing dorsal tracts. However, the action naming task appeared to be more sensitive for the visualization of the lexico-semantic tracts in the ventral stream. Further, Ntemou et al. propose the observation of transitivity in verb production. Ntemou details the kind of errors to be expected in a possible application to direct electrical stimulation (DES) procedures. In turn, Senova, and Ohlerth or Ntemou, offer complementary viewpoints. The last two papers provide specific linguistic functional information to explore using nTMS.

The proposal of Banjac et al. remarkably involves a combined assessment of two interrelated functions, language, and memory. The paper is also notable in the way data from a healthy sample and data from a sample of epileptic patients are contrasted, emphasizing brain reorganization. Furthermore, they successfully contrast individual and group data, demonstrating the feasibility of the proposed paradigms in clinical settings. On the other hand, Tomasino et al. exemplify the application of a protocol for real-time neuropsychological testing during left temporal tumor resection. Their protocol can be fully or partially applied, with language and memory assessed independently. The contrast between Banjac and Tomasino's proposals raises the question of a choice between synthetic or analytic testing procedures.

Two more papers involve the study of language in peri-surgical contexts. Using EEG, Nienke and collaborators suggest the prognostic value of an electrophysiological marker, namely, increased theta activity, in glioma patients. On the other hand, a detailed revision on the perioperative assessment of language in multilingual patients is included. De Martino et al. point to the current methodological heterogeneity concerning said assessment. Revision and advances are proposed in several dimensions: (1) linguistic distance between languages, (2) variables that determine the neural basis for each language (e.g., proficiency or age of acquisition), (3) language used for preoperative testing, and (4) used tasks during surgery. The authors propose very specific paths of improvement.

Finally, and going beyond language mapping, Nakajima et al. presented a work addressing attentional functions, through the color Stroop and cancelation tests. They mainly focus on the postoperative period, tracking it longitudinally: after surgery and 3 months later. Through a voxel-based lesion symptom mapping (VSLM), and establishing the lesions as the resection cavities, they show how the cingulate cortex (CC) zone II and the frontostriatal tract (FST), jointly explain prolonged selective attentional deficits. These data can be interesting for preoperative neurosurgical counseling (high risk of permanent impaired attention operating on CC zone II and FST) and for intra-operative neuropsychological strategy in awake surgery because working in that area with different cognitive tasks might not be reliable.

There is still much interdisciplinary work missing to efficiently link recent advances in cognitive neuroscience and all the facets of neurosurgery. A key aspect, in our opinion, involves the need for robust paradigms, sensitive at the single case level. This view might prove challenging and in contradiction with the proposed solutions to the current replication crisis (e.g., Turner et al., 2018). Those solutions imply, among other things, the insistence on very large sample sizes. However, to advance in the presurgical mapping of cognitive functions we cannot elude the mandatory direct applicability to the single case. Here we have presented attempts to link the group to the single case, and other recent efforts exist in the literature (Ekert et al., 2021).

Another way of advance includes pursuing specificity and sensibility in the tasks used during surgery (Gobbo et al., 2021). We have presented here some examples in the language paradigms that are more frequently used. Inspired by previous neurocognitive literature, an increment in task specificity might well require systematic attention to the emitted errors during DES stimulation upon given item characteristics (as in Della Puppa et al., 2013; Semenza et al., 2017; Gobbo et al., 2021). This, in turn, would impact on focality on an item-by-item basis: it would avoid false negatives and positives during the DES procedure. Finally, the presented contributions are mainly focused on language as has been the tendency in the past (see Ruis, 2018 for a recent systematic review). Ideally, we would need a task-to-brain atlas encompassing neural networks beyond language, that could be easily implemented and available to any surgical team.

## Author Contributions

All authors listed have made a substantial, direct, and intellectual contribution to the work and approved it for publication.

## Conflict of Interest

The authors declare that the research was conducted in the absence of any commercial or financial relationships that could be construed as a potential conflict of interest.

## Publisher's Note

All claims expressed in this article are solely those of the authors and do not necessarily represent those of their affiliated organizations, or those of the publisher, the editors and the reviewers. Any product that may be evaluated in this article, or claim that may be made by its manufacturer, is not guaranteed or endorsed by the publisher.
